# Supplementary material for: Impact of virus subtype and host IFNL4 genotype on large-scale RNA structure formation in the genome of hepatitis C virus
Source: RNA. 2020 Nov;26(11):1541–56. doi: 10.1261/rna.075465.120 (PMC7566573; doi:10.1261/rna.075465.120)
Supplement: Supplemental Material [file supp_26_11_1541__index.html]

Impact of virus subtype and host IFNL4 genotype on large-scale RNA structure formation in the genome of hepatitis C virus — Impact of virus subtype and host IFNL4 genotype on large-scale RNA structure formation in the genome of hepatitis C virus — Supplemental Material 

# Impact of virus subtype and host *IFNL4* genotype on large-scale RNA structure formation in the genome of hepatitis C virus

## Supplemental Material

- Supplemental\_Tables\_Figures.pdf
