# Supplementary material for: Impact of virus subtype and host IFNL4 genotype on large-scale RNA structure formation in the genome of hepatitis C virus
Source: RNA. 2020 Nov;26(11):1541–56. doi: 10.1261/rna.075465.120 (PMC7566573; doi:10.1261/rna.075465.120)
Supplement: Supplemental Material [file supp_075465.120_Supplemental_Tables_Figures.pdf]

SUPPLEMENTARY DATA

TABLE S1: GENBANK ACCESSION NUMBERS OF VIRUS SEQUENCES

FMDV-O: AY593824, JN998086, FJ461345, DQ248888, AY686687, EF552691, JX066664, JX040492, KF694737, AY593813, AY593822, KJ206910, KJ825804, AY593826, KF112882, HM191257, GU384683, HM008917, EU448369, AY593817, GU125647, AY593833, KF112880, KF112885, JX869177, HQ632769, AY593827, AF511039, AY333431, DQ478937, KF112888, FJ461344, AY593812, HQ412603, HQ113232, KJ206908, HQ632768, AY359854, FJ175664, KF112879, JQ900581, AY593830, AY317098, AY312588S2, GU125650, KF501486, HQ009509, AY593834, AF377945, DQ119643, KF112886, EU140964, HQ632770, KF501488, KM268895, HQ632771, JX869178, AY593825, JF749851, HQ632772, HQ268524, AY593823, AY593821, KF985189, KJ206909, JX040501, AY593828, AY593829, KJ825801, AB079061, AY593814,

HPgV-1 (n=100): AF121950, D87255, AF081782, D90600, LT009483, LT009487, LT009485, LT009494, AY196904, MH053119, LT009481, LT009478, HGU44402, LT009479, AF309966, MK291245, MK291244, AF031827, MH053115, KP259281, MH053120, HGU45966, MH053118, MH053121, HGU63715, LT009486, AB003289, LT009489, AF104403, JN127373, D90601, AB013501, MH053116, D87711, HGU36380, AB008335, D87263, D87712, D87713, KM670109, D87262, D87709, KM670096, LT009490, AB008342, KM670099, D87714, LT009480, KP710601, KM670110, LT009482, D87710, AB021287, KM670097, LT009484, KP710599, HQ331235, KM670100, HQ331234, LT009488, KM670107, HQ331233, KM670108, KP710604, KM670102, KM670106, KC618400, D87708, AB008336, KM670098, HGU94695, KP710605, KP710600, KC618401, MH746815, KC618399, AB013500, KP710598, KC618398, KM670101, MH053117, KP710602, KP710603, AB003288, KP710606, AB018667, HGU75356, AY949771, D87715, AB003290, AB003293, AF006500, AB003291, AB003292, MH179063, LT009493, MK291243, LT009492, LT009491, AF121950,

MNV (n=65): FJ446719, JN975495, EU004682, DQ223042, EU004668, AB435515, EU004680, JF320653, JN975498, EU004675, JF320647, DQ223043, EU004671, FJ446720, EU004674, EU004670, EU004681, EU004658, EU004677, HQ317203, EU004667, JN975493, JF320652, EU854589, JN975496, EF531290, AB601769, JN975494, JQ237823, EU004678, EU004663, DQ911368, EF531291, EU004683, EU004679, EU004664, DQ223041, EU004673, DQ285629,

EU004656, EU004659, EU004661, EU004662, AY228235, EU004655, EF014462, EU004654, EU004657, JF320648, JF320649, JF320650, JF320645, JF320651, JF320646, EU004669, EU004665, EU004672, EU004660, AB435514, JF320644, EU004676, JQ658375, EU004666,

FMDV-O (n=67): AY593824, JN998086, FJ461345, DQ248888, AY686687, EF552691, JX066664, JX040492, KF694737, AY593813, AY593822, KJ206910, KJ825804, AY593826, KF112882, HM191257, GU384683, HM008917, EU448369, AY593817, GU125647, AY593833, KF112880, KF112885, JX869177, HQ632769, AY593827, AF511039, AY333431, DQ478937, KF112888, FJ461344, AY593812, HQ412603, HQ113232, KJ206908, HQ632768, AY359854, FJ175664, KF112879, JQ900581, AY593830, AY317098, AY312588S2, GU125650, KF501486, HQ009509, AY593834, AF377945, DQ119643, KF112886, EU140964, HQ632770, KF501488, KM268895, HQ632771, JX869178, AY593825, JF749851, HQ632772, HQ268524, AY593823, AY593821, KF985189, KJ206909, JX040501, AY593828, AY593829, KJ825801, AB079061, AY593814,

HPeV-3(n=38): KX068679, KM986843, KJ659490, AB084913, AJ889918, KY020128, JX682576, GQ183029, GQ183033, GQ183032, GQ183031, GQ183026, GQ183027, KY556675, MK604044, MF371334, MK604049, KT879915, KT879922, KY351616, KY351618, JX826607, MF797924, MF797928, MF797929, KT626009, MF797935, MF797926, MF797925, MF797932, MF797916, MF797931, AB668029, LC129275, LC129276, LC129274, AB759205, AB759199,

JEV (n=65): JN381838, KT957421, JN381872, JN381830, JQ086763, JN381834, AF075723, HQ652538, AB241119, JN381867, AY184212, AB551990, AB569988, AF217620, KM658163, AB471669, AY303796, AB051292, KY927815, KF297916, HQ223286, M18370, JF706283, JN381853, AF080251, KY927818, AY316157, GQ902063, JF915894, KY078829, JN381863, AB551991, AF045551, KT229572, L48961, HQ223287, JN381843, JF706270, HQ223285, GQ902062, GQ902061, KM677246, KT957420, AY303794, KT229575, JF706279, HE861351, JN381868, JN381870, KY927816, U47032, AF098736, EF623987, JN381873, AB241118, JN381848, JN381866, AF221500, AY303792, GU205163, KF667311, GQ902060, JN381851, KU351667, EU429297,

EV-A71 (n=238): MK652139, DQ341367, MG976581, KX372324, JX244184, MH716347, JQ639384, LT719065, LC506514, KC436271, MG214681, KT428646, KP289432, MH716380, JF738002, HQ647173, GQ279369, KU936120, HM807310, FJ607337, FJ357384, MG367595, LC321989, MH716337, AB575917, KX372322, KJ004560, KF982854,

JQ074189, MG367594, EU131776, LT719063, LT719067, AB575918, MN254979, MF662695, KJ400360, U22522, KJ686246, KU936132, AB575938, HM002487, HQ647172, KJ686249, JN992284, KC436265, AB575935, KU936128, FJ194964, KJ784495, AB747375, JX678885, DQ341358, LC375764, AB575928, MF973167, LC506513, FJ357376, LK985324, MG672479, KF142413, MG773126, KX372317, KP289419, KP289425, KP289422, AB575911, KT345959, MG672481, GQ994992, DQ341364, DQ341357, KU936121, KC436266, KY612315, KC109780, AB575914, GQ231939, MH111073, JQ681218, KJ686308, MH716310, AF302996, GQ994989, KY315729, AM396584, LT719064, KM055005, KU254596, KP308450, HQ456306, KC436270, MG367600, KU936125, JQ742001, KC954664, FJ607336, HQ189392, KT008669, DQ341355, JQ742002, KX372328, JQ280307, KX372310, KM673244, DQ341359, KT428647, AM396587, KJ686262, FJ606447, KP289431, KX197462, AB575936, MG672478, LT719068, KP289424, EF063152, MG207962, EU414333, FJ360545, AF316321, JF738001, MF662701, KJ686208, KP308426, JF799986, MH484070, KF974790, GQ231934, U22521, AB575923, GQ994991, MG431943, JQ316638, DQ341360, KX372308, AY465356, HQ647179,

HCV all genotypes (n=125): EF108306, AF009606, D90208, D14853, KJ439768, KC248194, AM910652, KC248198, KJ439772, KC248193, KJ439778, KJ439781, KJ439774, HQ537007, AJ851228, KC248195, KJ439780, KJ439779, KJ439776, KJ439777, D00944, D10988_D01221, D50409, JF735114, JF735120, KC844042, DQ155561, HM777358, AB031663, JF735111, FN666428, JF735115, KC197238, JF735112, JF735116, JF735118, JF735117, JF735119, JF735110, KC197236, KC197237, KC197239, D17763, D49374_D26556, KJ470619, KJ470618, JX227954, JF735121, FJ407092, D63821, JF735124, Y11604, FJ462436, DQ418786, EF589161, FJ462432, EU392173, FJ839870, FJ462433, FJ462441, FJ462440, FJ462431, FJ462434, FJ462439, JF735136, FJ839869, HQ537009, HQ537008, FJ025855, FJ025854, JX227964, JF735127, JF735132, JF735131, JF735130, JF735129, JF735138, JF735135, JF735134, AF064490, Y12083, D84262, EF424629, D84263, DQ314805, DQ835760, D63822, D84265, DQ835770, DQ835761, D84264, EF424628, DQ835767, DQ278894, EF424627, EF424626, EF424625, EU408328, EU408329, EF632071, EU246940, EU158186, DQ278892_AY834954_AY835335, EU643836, EU408330, JX183552, KJ567651, KM252789, JX183557, DQ278891, DQ278893, JX183558, JX183553, JX183554, JX183551, JX183549, JX183550, KJ470620, KJ470621, KJ470622, KJ470623, KJ470624, KJ470625, KC844039, KC844040,

TABLE S2

LIST OF CONTROL SNPs

Chr	Position	rs_ID	Orient
chr4	12983343	rs10000091	+
chr1	111734975	rs1000947	+
chr4	63197594	rs10010596	+
chr4	100695124	rs10030146	+
chr4	183534161	rs10033074	+
chr4	114323981	rs10034750	+
chr9	34631162	rs10046843	+
chr4	23288098	rs10050029	+
chr8	26285479	rs10093390	+
chr8	28228291	rs10094977	+
chr20	38732301	rs1010321	+
chr8	60948037	rs10108543	+
chr8	118714003	rs10113233	+
chr9	108381380	rs10115131	+
chr7	143452257	rs1014069	-
chr14	44561671	rs10149946	+
chr2	32479118	rs10182170	+
chr2	151625463	rs10186482	+
chr2	235032893	rs10190447	+
chr7	34445525	rs10241710	+
chr7	151127257	rs10256812	+
chr7	50273756	rs10276619	+
chr6	24961163	rs1028477	-
chr10	130892388	rs1036270	+
chr22	37283722	rs1041895	+
chr6	55145701	rs10456701	+
chr5	167421729	rs10475843	+
chr5	147809331	rs10477350	+
chr7	142469229	rs10487531	+
chr9	27468463	rs10511816	-
chr9	87555970	rs10512187	+
chr12	125025558	rs1055472	+
chr1	84647546	rs1057746	-
chr10	78299615	rs10762779	+
chr12	125726715	rs10773198	+
chr11	134901914	rs10791403	+
chr10	7904008	rs10795572	+
chr1	30105771	rs10799077	+
chr7	129977887	rs10808241	+
chr9	34596680	rs10814125	+
chr11	48560213	rs10838956	+
chr12	3218420	rs10848829	+
chr12	80057464	rs10862038	+
chr1	9388590	rs10864413	+
chr9	69204747	rs10870017	+
chr10	112662640	rs10885369	+
chr10	117770266	rs10886131	+
chr1	55292134	rs10888912	+
chr11	115233890	rs10891814	+
chr5	139396890	rs10900862	+
chr12	131620248	rs10902530	+
chr10	123066903	rs10902870	+
chr10	14671222	rs10906740	+
chr10	18964758	rs11007866	+
chr10	128962749	rs11016572	+
chr11	48761945	rs11040016	+
chr17	6374568	rs11078621	+
chr21	25965946	rs11087985	+
chr12	95596979	rs11108147	+
chr1	210824120	rs11119611	+
chr20	23343350	rs1112819	+
chr16	74943972	rs11149785	+
chr1	40515059	rs11208299	+
chr2	18902959	rs1122624	-
chr8	10393755	rs11249999	+
chr10	6288034	rs11257854	+
chr12	122327956	rs1129167	-
chr11	134294724	rs1144221	-
chr14	55845400	rs11621920	+
chr14	104494153	rs11626836	+
chr15	73883936	rs11639300	+
chr16	88078091	rs11639845	+
chr16	31410096	rs11640148	+
chr16	51524634	rs11646208	+
chr17	4453080	rs11655342	+
chr2	18338468	rs11689614	+
chr4	168047067	rs11728448	+
chr4	16994880	rs11731316	+
chr4	179752670	rs11736914	+
chr4	187969824	rs11737485	+
chr5	50616675	rs11746825	+
chr6	70102381	rs11759810	+
chr7	12556990	rs11764174	+
chr1	157163842	rs1176544	+
chr8	122795139	rs11775943	+
chr10	117148539	rs11819305	+
chr14	103970594	rs11851097	+
chr16	80669860	rs11863297	+
chr9	84701953	rs1187336	-
chr3	193956972	rs11914490	+
chr3	172584145	rs11922343	+
chr1	220500441	rs12120994	+
chr1	34325265	rs12130968	+
chr1	91570783	rs12141173	+
chr21	21147102	rs12152089	+
chr2	212735163	rs12185565	+
chr6	44494493	rs12192702	+
chr1	247438293	rs12239046	+
chr12	16124240	rs12297516	+
chr14	49490614	rs12323543	+
chr11	4848419	rs12361955	+
chr11	48534405	rs12365220	+
chr14	53694533	rs12433844	+
chr17	37137586	rs12450937	+
chr3	169602805	rs12487498	+
chr6	140866118	rs12524925	+
chr6	55479176	rs12526863	+
chr7	118757867	rs12536552	+
chr8	80421136	rs12545933	+
chr9	80192588	rs12554086	+
chr15	67103580	rs12594610	+
chr1	214836031	rs1261868	-
chr4	61235270	rs12639891	+
chr5	175865617	rs12652846	+
chr6	144202524	rs12664476	+
chr8	135820613	rs12678843	+
chr7	113677653	rs12705891	+
chr12	14510823	rs12708367	+
chr1	190021748	rs12732394	+
chr1	38962356	rs12733433	+
chr3	59676390	rs1281463	+
chr12	94754980	rs12822392	+
chr15	54465639	rs12904540	+
chr17	72996427	rs12947636	+
chr18	45660009	rs12957040	+
chr2	119112657	rs13009596	+
chr21	45347113	rs13049248	+
chr3	194698992	rs13084683	+
chr4	101210521	rs13130412	+
chr4	142689140	rs13133181	+
chr5	154408936	rs13162143	+
chr5	12013356	rs13171948	+
chr5	29952825	rs13183145	+
chr5	3855257	rs1319960	+
chr8	71089696	rs13251367	+
chr8	126268783	rs13259816	+
chr8	18658330	rs13274103	+
chr9	32205706	rs1331240	+
chr16	74538674	rs13336493	+
chr2	170214402	rs13385825	+
chr13	87501880	rs1340833	+
chr22	49532624	rs134452	-
chr16	11585967	rs1345441	-
chr7	54349136	rs1357884	-
chr5	156956411	rs1363232	-
chr18	31207561	rs1365294	-
chr9	78928950	rs1371013	-
chr13	60391353	rs1390157	+
chr3	28917720	rs1392307	+
chr12	99476849	rs1398084	+
chr2	64558019	rs1421337	+
chr11	131030204	rs1433982	+
chr4	108843849	rs1452688	-
chr15	94981210	rs1457851	-
chr10	60314381	rs1471246	+
chr12	25065406	rs1497249	-
chr2	57476332	rs1518685	-
chr2	166335304	rs1528487	-
chr10	2397288	rs1532215	+
chr21	38987079	rs1537106	+
chr16	82529949	rs1560824	-
chr8	4844711	rs1561360	+
chr17	73242948	rs1566290	+
chr6	32701241	rs1612904	-
chr17	18930497	rs1634411	-
chr10	82125138	rs1649934	-
chr1	229171169	rs16849500	+
chr8	40639459	rs16889787	+
chr1	150319969	rs1694379	-
chr16	82642261	rs16957935	+
chr16	25067419	rs16974327	+
chr8	4247625	rs1714746	-
chr5	98319582	rs17165849	+
chr4	73595110	rs17192483	+
chr14	59251812	rs17255451	+
chr17	18937120	rs1737944	-
chr2	73695962	rs17434655	+
chr11	61888645	rs174455	+
chr15	77368003	rs17470228	+
chr10	29949908	rs1756752	-
chr15	51397575	rs17648128	+
chr15	51402295	rs17648140	+
chr14	38945690	rs17670531	+
chr21	41898747	rs177933	+
chr18	73625792	rs17829298	+
chr21	24122423	rs1783355	+
chr18	13116433	rs1786263	-
chr14	30858598	rs179666	-

chr7	44834514	rs1802437	-
chr2	236933481	rs1821655	+
chr3	103875711	rs1824240	+
chr5	58907240	rs184187	+
chr3	72500964	rs1898634	+
chr4	131514178	rs1911889	+
chr12	114268404	rs1920591	+
chr5	30870751	rs1921111	+
chr5	136700906	rs193491	-
chr10	109646537	rs1936138	-
chr11	98269834	rs1941784	-
chr7	29493778	rs1946134	+
chr2	227198074	rs1950135	-
chr14	32417836	rs1957007	+
chr6	54908016	rs1963697	-
chr6	7922058	rs197113	+
chr2	73700971	rs2001490	+
chr1	17071209	rs2016693	-
chr18	68729625	rs2048329	+
chr3	150914987	rs2061201	-
chr6	98946786	rs2064146	-
chr14	103412850	rs2065018	-
chr22	32435553	rs2076050	+
chr2	85668215	rs2077079	-
chr2	236945804	rs2082870	+
chr4	53564319	rs2099453	+
chr3	46561246	rs2139633	+
chr20	10847619	rs2144186	-
chr6	18207102	rs214600	-
chr10	118951825	rs2148496	-
chr1	241480284	rs2153693	+
chr7	32297831	rs215606	+
chr2	77924959	rs2163875	-
chr14	32491287	rs2180866	+
chr10	36774764	rs2185863	-
chr12	11702690	rs2187642	+
chr14	80016476	rs2193131	+
chr12	99234519	rs2201160	+
chr2	235335632	rs2217538	-
chr16	31363214	rs2230429	+
chr22	37066886	rs2235321	+
chr17	82675394	rs2248371	+
chr8	11853550	rs2272767	+
chr15	72735137	rs2277598	-
chr14	64796582	rs229587	+
chr1	206474694	rs2297546	-
chr1	119383313	rs2298028	+
chr11	20638211	rs2298826	+
chr16	661905	rs2301426	-
chr17	5532943	rs2301582	-
chr17	40935654	rs2315602	+
chr22	24602652	rs2330805	+
chr9	30077007	rs2360147	+
chr13	103960244	rs2391059	+
chr10	25750132	rs2442855	-
chr12	48793667	rs2446990	+
chr11	27379170	rs2472625	-
chr14	88267790	rs2474952	-
chr1	2205929	rs2503712	+
chr3	135050073	rs253876	-
chr5	172460777	rs2569245	+
chr5	75742893	rs258494	+
chr16	61089243	rs2639889	-
chr5	115842698	rs26538	+
chr5	115229045	rs265436	+
chr16	78547266	rs2667649	+
chr4	74011427	rs2668076	+
chr14	99180992	rs2693680	+
chr11	75147924	rs2712806	+
chr5	96384871	rs271942	-
chr5	60456813	rs27223	+
chr1	3122059	rs2742672	-

chr4	3718102	rs2748777	-
chr2	32557696	rs2754513	+
chr1	57218388	rs2764660	+
chr9	69463950	rs2781538	+
chr13	97682683	rs2783482	+
chr10	117436739	rs2794424	+
chr12	95428932	rs280965	+
chr1	72346757	rs2815752	-
chr5	75745736	rs28225	-
chr21	21133424	rs2826674	+
chr16	77555105	rs282985	+
chr5	142108414	rs28372841	+
chr12	132427238	rs28386864	+
chr6	62385346	rs2842783	-
chr6	31365526	rs2844580	-
chr12	132427669	rs28451398	+
chr20	57491781	rs2865387	+
chr5	51023481	rs28654104	+
chr7	212512	rs28706381	+
chr17	36887732	rs2898660	-
chr4	56179091	rs2899056	+
chr8	17155132	rs2904659	+
chr5	76272228	rs2937715	-
chr1	84582958	rs2945152	-
chr12	56720523	rs2958127	-
chr16	9153026	rs2965895	-
chr12	52424720	rs298104	-
chr6	56746538	rs3002005	+
chr3	11749236	rs301554	+
chr18	50448374	rs3017261	+
chr2	230982495	rs3106082	+
chr7	135702747	rs3112346	+
chr6	29875102	rs3115619	-
chr14	58672348	rs311845	+
chr10	113645856	rs3127106	+
chr6	29872871	rs3132714	-
chr6	29868800	rs3132718	-
chr12	56725452	rs3214051	-
chr12	62763403	rs342148	+
chr10	69324130	rs34348007	+
chr18	38898108	rs34434760	+
chr13	42214119	rs347418	+
chr17	7365448	rs34755955	+
chr8	65191690	rs34994298	+
chr20	17927493	rs35016526	+
chr1	212276874	rs351417	-
chr20	60341506	rs354741	+
chr7	65068922	rs35794351	+
chr12	112630967	rs36030379	+
chr3	114236317	rs3732782	-
chr1	117100373	rs3754104	-
chr17	68206938	rs3760274	-
chr20	3795528	rs3761218	+
chr1	8974362	rs3765964	-
chr1	8951527	rs3765968	-
chr1	201509148	rs3767525	-
chr15	101391959	rs3784481	-
chr20	17466709	rs3790338	-
chr14	44506307	rs3809430	-
chr1	17071812	rs3818048	-
chr7	15873963	rs38205	+
chr4	902698	rs3822023	-
chr5	41573733	rs3863154	-
chr5	103674293	rs3909338	-
chr1	36480052	rs3917924	-
chr6	98785572	rs3923049	+
chr3	192230660	rs3925222	-
chr9	130409635	rs3926856	-
chr5	31682580	rs39791	+
chr1	62221432	rs4019803	-
chr20	45751708	rs405247	-
chr6	24803597	rs4074236	-

chr22	24010332	rs422674	+
chr3	124242241	rs4234217	+
chr11	72571781	rs426117	-
chr17	3036288	rs4264423	+
chr5	107058155	rs4269278	+
chr4	132737074	rs4373171	+
chr2	136689878	rs4419275	+
chr11	61549915	rs441938	-
chr12	12879853	rs4462437	+
chr10	34905460	rs4469833	+
chr6	74073586	rs4495244	+
chr9	8225531	rs4497021	+
chr7	22112350	rs4598147	+
chr16	82799344	rs4598906	+
chr1	182140796	rs4652689	+
chr2	19791948	rs4666348	+
chr6	31195186	rs4713447	+
chr7	64991278	rs4718177	+
chr7	23789469	rs4722272	+
chr9	69009361	rs4745500	+
chr12	130117883	rs4760065	+
chr6	163118257	rs476653	+
chr13	106406156	rs4772781	+
chr15	93382098	rs4777857	+
chr17	77595906	rs4789469	+
chr17	55304435	rs4793788	+
chr20	24633595	rs4813525	+
chr2	18824642	rs4832456	+
chr1	151809931	rs4845587	+
chr2	69115401	rs4854547	+
chr9	86657853	rs4878000	+
chr16	77701577	rs4887918	+
chr14	101174641	rs4900487	+
chr6	11621871	rs490280	+
chr15	25558943	rs4906971	+
chr1	18852610	rs4920564	+
chr2	44096960	rs4952699	+
chr3	187845171	rs503787	+
chr1	115494175	rs506049	+
chr6	106369142	rs510405	-
chr18	77250689	rs5374	+
chr4	9800648	rs56139970	+
chr10	101280781	rs56171414	+
chr16	88281952	rs56302546	+
chr3	9556313	rs56407147	+
chr2	54692088	rs56898160	+
chr22	37071352	rs5756506	+
chr11	76172117	rs57946456	+
chr12	10733168	rs5796416	+
chr13	36928460	rs582091	-
chr3	3125400	rs59286216	+
chr4	47999036	rs59363169	+
chr22	45051049	rs6007434	+
chr3	8760830	rs60345038	+
chr20	88453	rs6039403	+
chr20	2558741	rs6050063	+
chr20	35006423	rs6060151	+
chr20	32004423	rs6061087	+
chr20	48149399	rs6066644	+
chr20	42344620	rs6102788	+
chr1	199407986	rs612795	+
chr8	86092687	rs61629815	+
chr15	26976494	rs61999630	+
chr21	31590487	rs62222920	+
chr4	65731761	rs62299607	+
chr4	175232180	rs62337024	+
chr7	143418968	rs62472729	+
chr7	143432213	rs62474772	+
chr1	233618245	rs635698	-
chr11	117293108	rs638405	+
chr11	78153964	rs643430	+
chr3	114236473	rs6438191	+

chr3	116015687	rs6438296	+
chr3	125009782	rs6438869	+
chr3	45124731	rs6441891	+
chr8	33498556	rs6468171	+
chr8	65178463	rs6472179	+
chr10	18902718	rs6481583	+
chr15	66957020	rs6494621	+
chr16	27813510	rs6498038	+
chr12	120764559	rs6553	+
chr5	165709385	rs6556822	+
chr12	120878449	rs656933	-
chr14	80457125	rs6574576	+
chr11	100069001	rs6590574	+
chr1	4459129	rs662036	-
chr2	39162037	rs66771682	+
chr1	159883032	rs6682796	+
chr4	115302551	rs66997216	+
chr6	116250092	rs6755	-
chr3	54601810	rs6765694	+
chr3	39847010	rs6777708	+
chr3	157407795	rs6803628	+
chr4	119025980	rs6828669	+
chr4	179766847	rs6844851	+
chr5	178538695	rs688074	+
chr6	125316311	rs6899690	+
chr6	1137899	rs6904803	+
chr6	36290890	rs6914153	+
chr6	131145967	rs6942184	+
chr7	18193559	rs6945251	+
chr7	15629375	rs6958841	+
chr12	130073404	rs697792	+
chr9	78927489	rs7030136	+

chr9	90808223	rs7036417	+
chr9	11039639	rs7044282	+
chr10	3616727	rs705469	+
chr10	129833518	rs7069546	+
chr2	154370165	rs707040	+
chr10	126163546	rs7077301	+
chr10	12547151	rs7078960	+
chr11	48489102	rs7103557	+
chr8	17313520	rs71212685	-
chr11	12992289	rs7129181	+
chr11	111185891	rs7130452	+
chr15	78841397	rs7166501	+
chr15	33213807	rs7169713	+
chr16	12442437	rs7185322	+
chr16	72090258	rs7190995	+
chr16	79115745	rs7191506	+
chr16	78590110	rs7200682	+
chr16	2013618	rs7200747	+
chr17	73535958	rs7209879	+
chr17	67029895	rs7216886	+
chr18	7067653	rs7234111	+
chr18	78440167	rs7241158	+
chr14	89727083	rs72689472	+
chr1	202264291	rs727724	+
chr6	155582297	rs72994676	+
chr12	63621661	rs7311307	+
chr13	39346149	rs7318557	+
chr13	74345518	rs7333414	+
chr13	103977322	rs7338623	+
chr16	29230794	rs735645	-
chr7	10888515	rs7357330	+
chr8	70524140	rs7387249	+

chr11	48497341	rs7395662	+
chr6	9413276	rs742511	-
chr1	25875935	rs74378533	+
chr5	175331195	rs7445817	+
chr2	225173107	rs751264	+
chr1	230452281	rs7514115	+
chr1	247599026	rs7523061	+
chr1	49017536	rs7524637	+
chr1	21514216	rs7535497	+
chr1	47247503	rs7552123	+
chr2	238927379	rs7571467	+
chr2	64538340	rs7581658	+
chr17	34606024	rs758409	-
chr2	46746261	rs7584870	+
chr3	77472155	rs7614439	+
chr20	23446011	rs761724	-
chr3	7469204	rs7623055	+
chr3	101546979	rs7628681	+
chr3	60116459	rs7648665	+
chr6	51748379	rs765525	-
chr2	73151113	rs768851	+
chr4	41798742	rs7689428	+
chr4	4689125	rs7692353	+
chr5	144145201	rs7716084	+
chr6	161778667	rs7754162	+
chr8	31898373	rs776378	+
chr6	69755811	rs7775172	+
chr7	133120296	rs7777197	+
chr7	138632991	rs7792330	+
chr7	12551849	rs7800736	+
chr8	139979861	rs7837321	+
chr9	1170227	rs7848270	+

FIGURE S1

GENOME SCANS OF Z-SCORES, MFED DIFFERENCES AND SEQUENCE DIVERGENCE

OF HCV GENOTYPES 1a, 1b AND 3a

A)

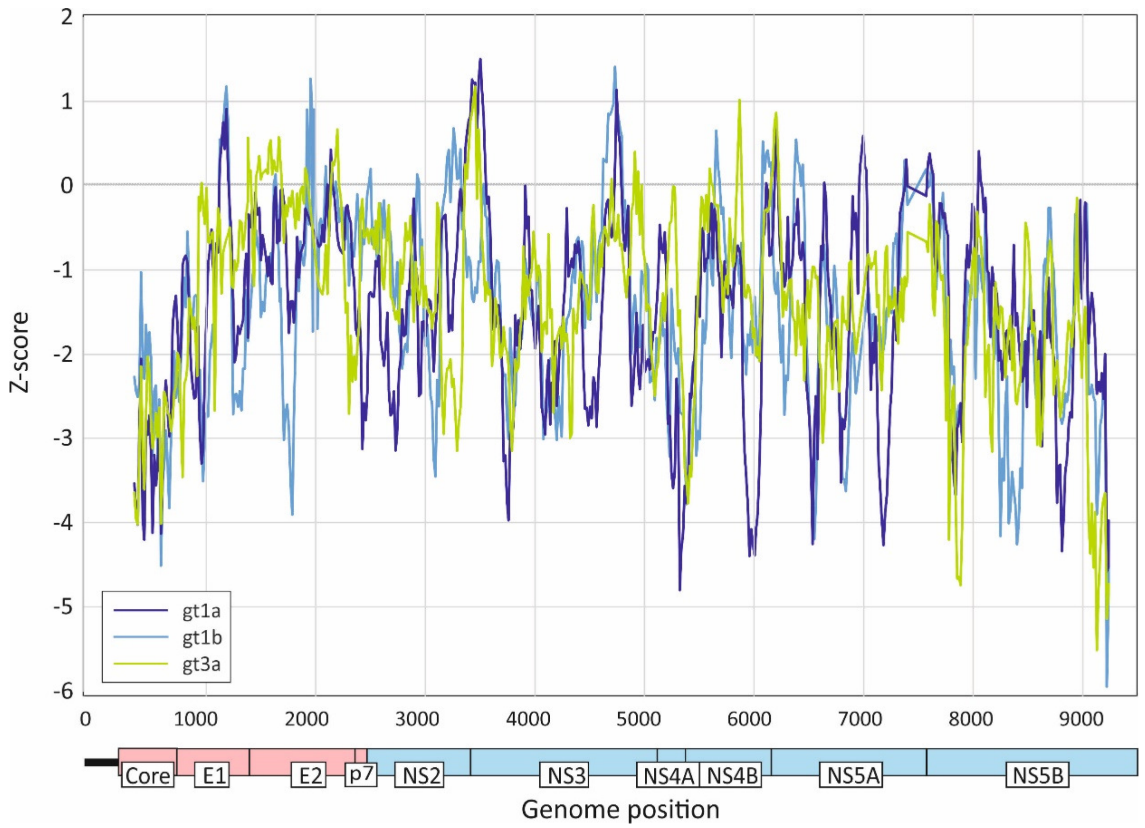

B)

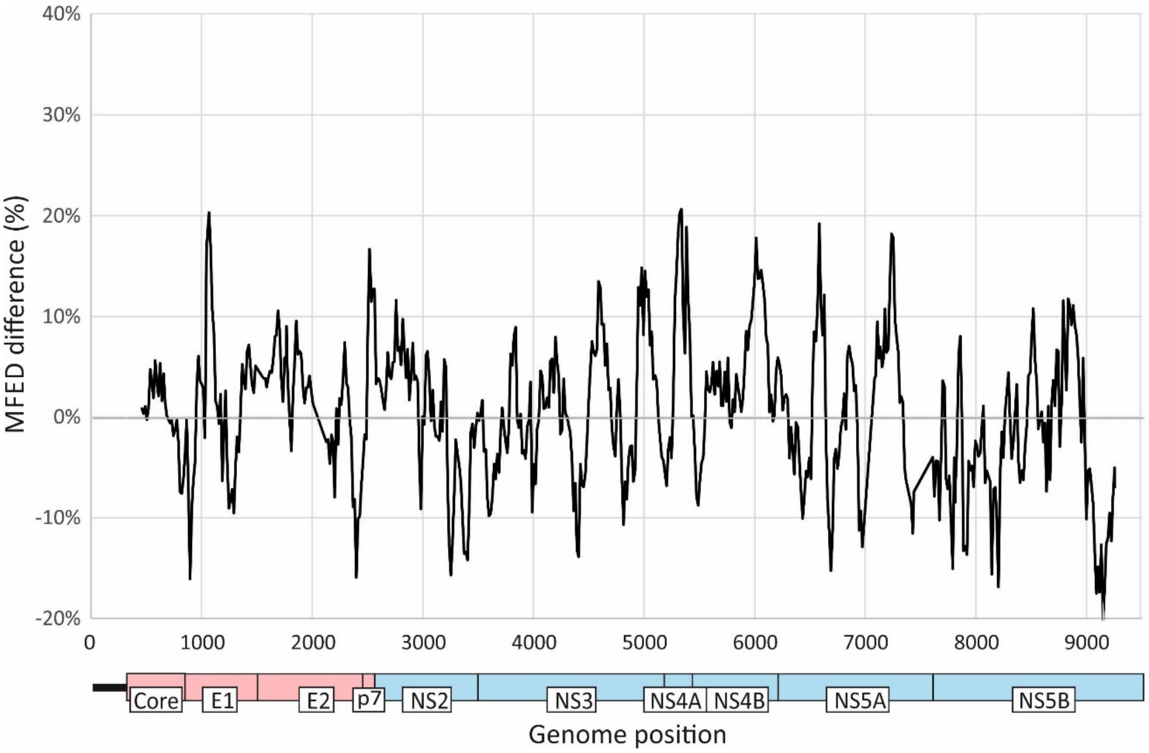

C)

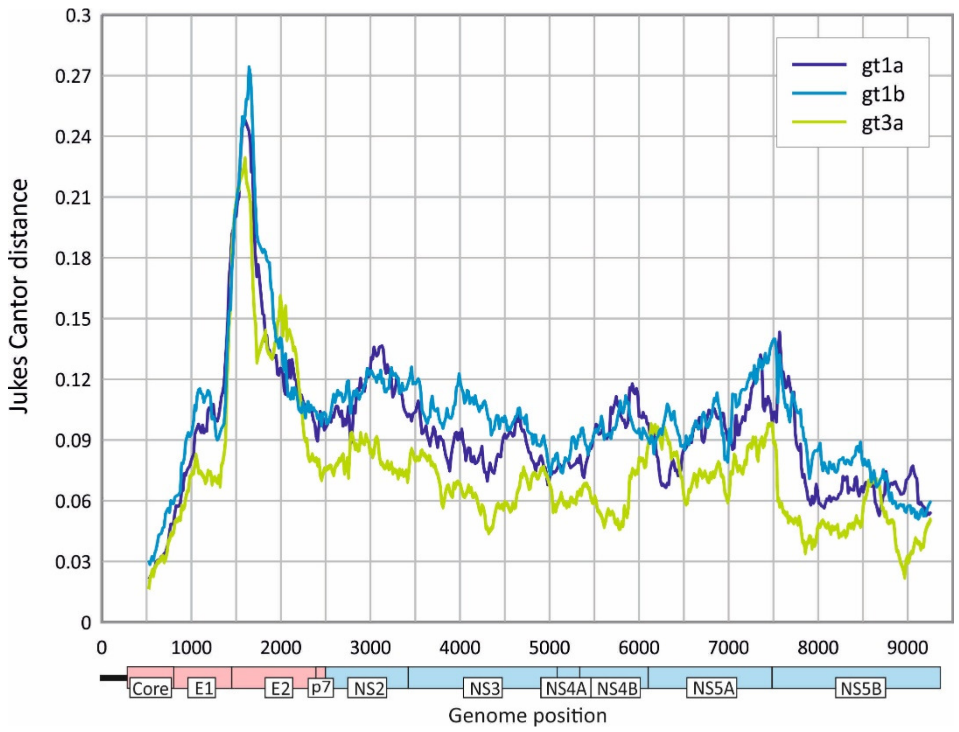

A) Z-scores calculated for consecutive 240 base fragments, incrementing by 15 bases across the HCV coding region (569 fragments). Z-scores represent the position of the MFE of the native sequence within the distribution of MFE values of 49 sequence order randomised controls. Graph lines show mean values of genotypes 1a (n=388), 1b (n=106) and 3a (n=855) sequences plotted by genome position. (B) Differences in MFED values for gt1a and gt3a scanned across the genome. (C) Mean divergence between prototype sequences of genotype 1a (M67463 – H77), 1b (D90208 – HCV-J) and 3a (D17763 – NZL-1) and representative sequences of each genotype sampled from the study population. Mean distances were calculated for 360 fragments incrementing by 15 bases between fragments with Jukes-Cantor correction for multiple substitutions. .

FIGURE S2

INFLUENCE OF SEQUENCE LENGTHS and SUBOOPTIMAL SAMPLING METHOD ON CONTOUR PLOT PREDICTIONS

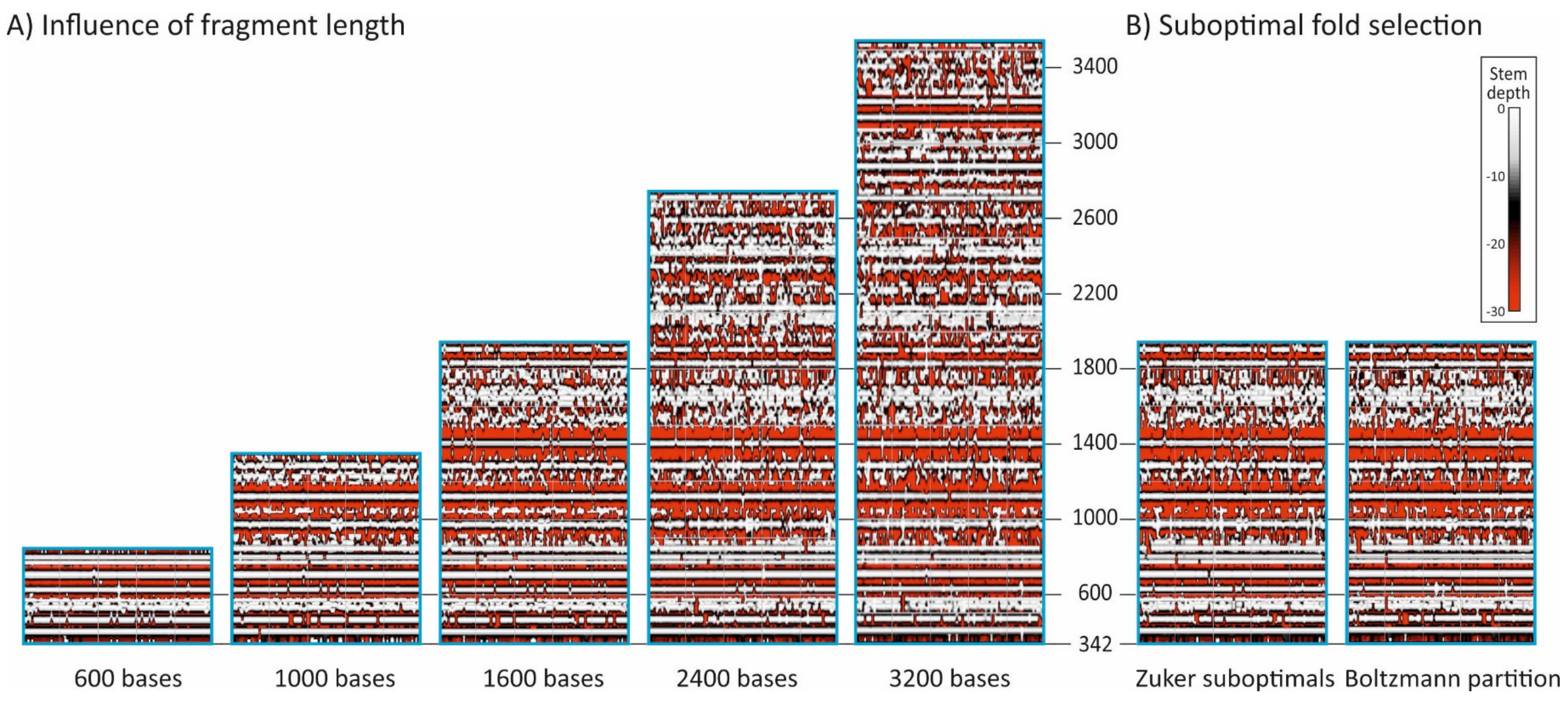

Contour plots of 99 HCV genotype 1b sequences generated using different run parameters. (A) Comparison of contour plots for sequence fragments of lengths 600, 1000, 1600 (as used for other plots presented in the study), 2400 and 3200 bases (approaching the upper bound of sequence length for RNAsubopt). (B) Comparison of the default selection methods for suboptimal folds (those within 5% of the optimum (minimum) MFE (selection of --deltaEnergy parameter in RNAsubopt) sd used in the plots shown in Fig. S2A) with alternative methods based on a random selection of Boltzman weights in the partition function (--stochBT) and on Zuker sub-optimals (--zuker).

FIG. S3. CONTOUR PLOTS OF PROTOTYPE SEQUENCES OF EACH DIFFERENT GENOTYPE AND SUBTPYE OF HCV

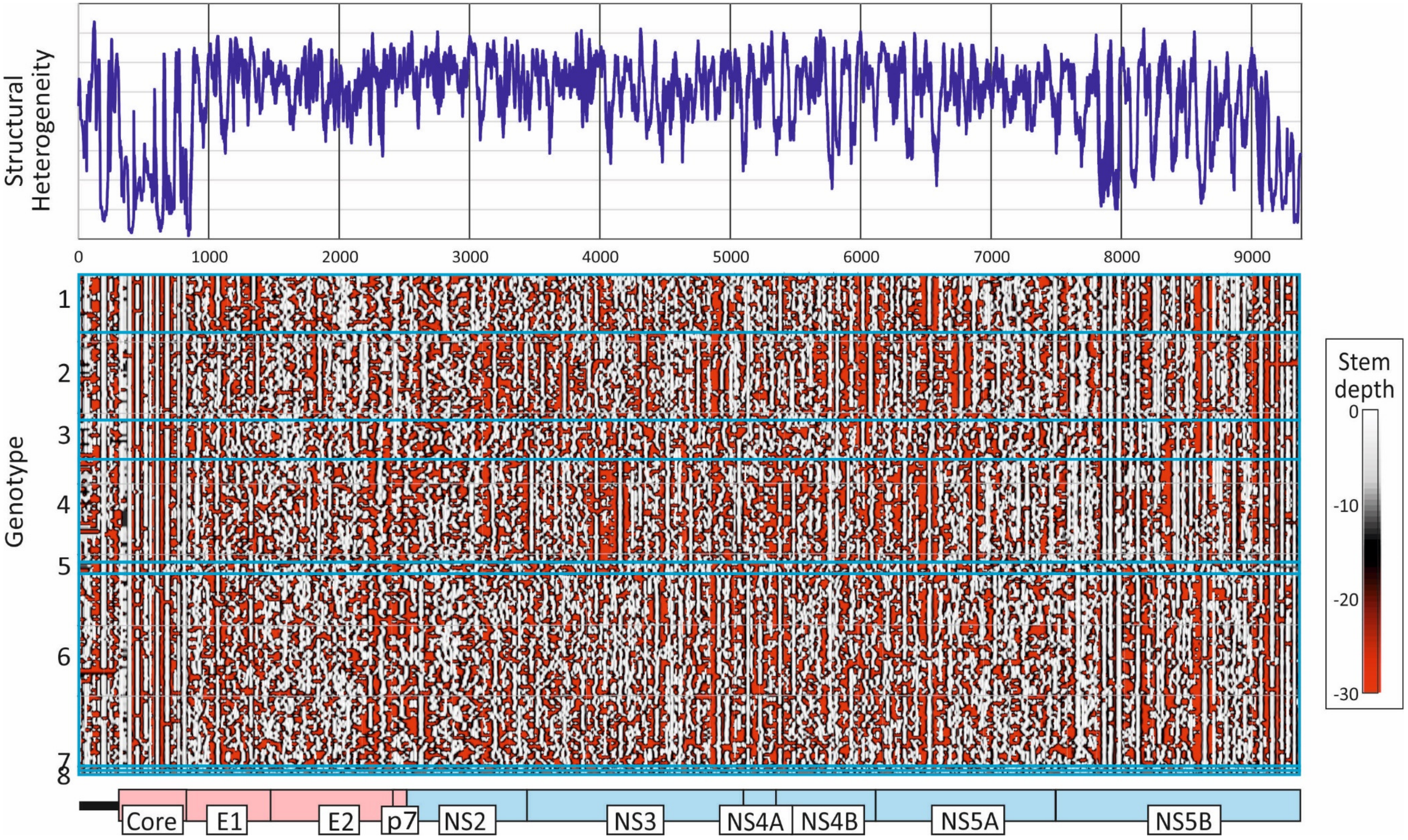

Contour plot of single examples of each currently classified and candidate HCV subtype and genotype (data derived from https://talk.ictvonline.org/ictv:wikis/flaviviridae/w/sg:_flavi/56/hcv-classification) listed in Table S1; Suppl./ Data.

FIGURE S4

MFED VALUES FOR REPRESENTATIVE EXAMPLES OF EACH HCV GENOTYPE / SUBTYPE

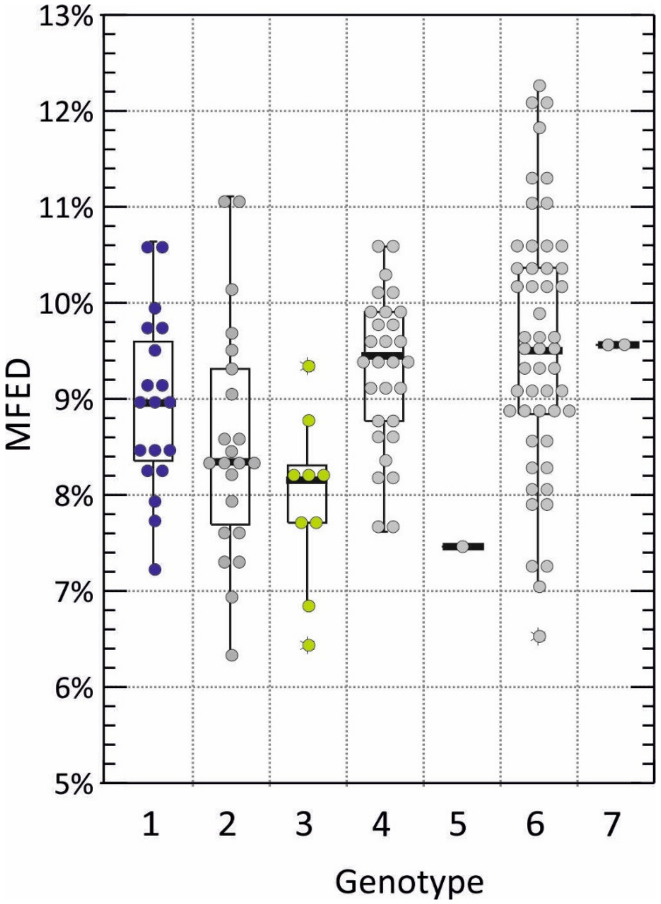

Mean values of sequence fragments for individual polyprotein sequences of all classified subtypes for HCV genotypes 1-7 (individual representative sequences for each subtype shown. The box plots show (from the top): 2 standard deviations (SDs) above mean, 1 SD above mean, mean, 1 SD below mean and 2 SDs below mean; stars represent outliers outside this range.

FIG. S5. CONTOUR PLOTS OF DIFFERENT FMDV TYPES

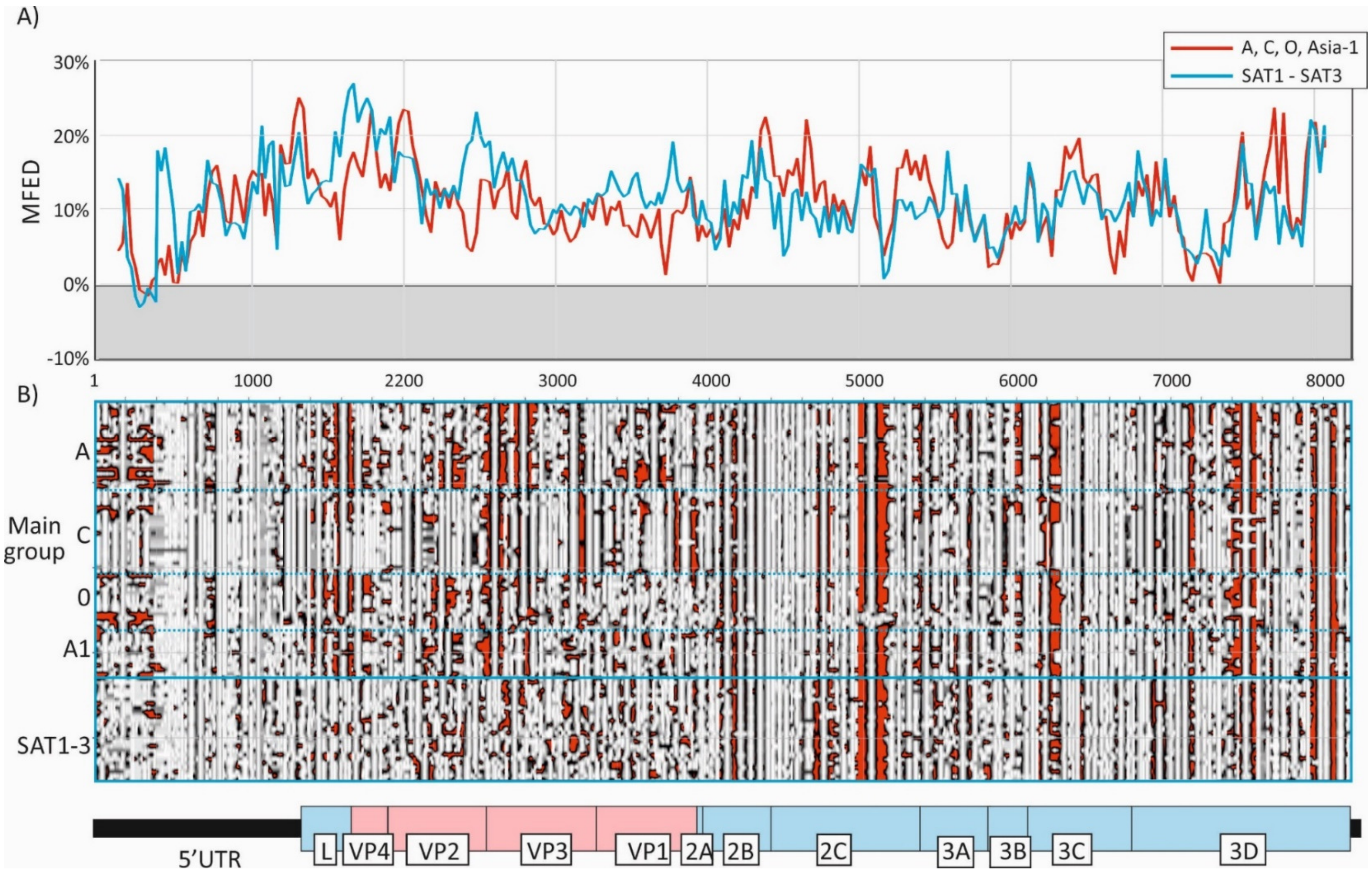

RNA structure prediction in different serotypes of FMDV. (A) MFED scan of the genome of the two FMDV groups using fragment sizes and increments as used for HCV (Fig. 1). (B) Contour plot of representative example sequences of each FMDV type.
